# How to improve the quality of care for women with postpartum haemorrhage at Onandjokwe Hospital, Namibia: quality improvement study

**DOI:** 10.1186/s12884-019-2635-6

**Published:** 2019-12-11

**Authors:** Tshimanga Nsangamay, Robert Mash

**Affiliations:** Division of Family Medicine and Primary Care, Stellenbosch University, Stellenbosch, South Africa

**Keywords:** Postpartum haemorrhage, Quality of care, Maternal mortality, Emergency care, Namibia

## Abstract

**Background:**

Postpartum haemorrhage (PPH) is the leading direct cause of maternal morbidity and mortality worldwide. The sustainable development goals aim to reduce the maternal mortality ratio to 70 per 100,000 live births. In Namibia, the ratio was reported as 265 per 100,000 live births in 2015 and yet little is published on emergency obstetric care. The majority of deliveries in Namibia are facility-based. The aim of this study was to assess and improve the quality of care for women with PPH at Onandjokwe Hospital, Namibia.

**Methods:**

A criterion-based audit cycle in all 82 women with PPH from 2015 using target standards for structure, process and outcomes of care. The audit team then planned and implemented interventions to improve the quality of care over a 10-month period. The audit team repeated the audit on all 70 women with PPH from the same 10-month period. The researchers compared audit results in terms of the number of target standards achieved and any significant change in the proportion of patients’ care meeting the predetermined criteria.

**Results:**

In the baseline audit 12/19 structural, 0/9 process and 0/3 outcome target standards were achieved. On follow up 19/19 structural, 6/9 process and 2/3 outcome target standards were met. There was one maternal death in the baseline group and none in the follow up group. Overall 6/9 process and 2/3 outcome criteria significantly improved (*p* <  0.05) from baseline to follow up. Key interventions included training of nursing and medical staff in obstetric emergencies, ensuring that guidelines and standard operating protocols were easily available, reorganising care to ensure adequate monitoring of women postpartum and ensuring that essential equipment was available and functioning.

**Conclusion:**

The study demonstrates that the quality of care for emergency obstetrics can be improved by audit cycles that focus on the structure and process of care. Other hospitals in Namibia and the region could adopt the process of continuous quality improvement and similar strategies.

## Background

Postpartum haemorrhage (PPH) is the leading direct cause of maternal morbidity and mortality worldwide [1, 2]. The condition contributes to 20% of maternal mortality globally and affects 2% of all women who give birth [3–5].

The Maternal Mortality Ratio (MMR) is an important indicator of the maternal health status within a given population [6]. The MMR in many African countries (510 per 100,000 live births for Africa in 2013) is very high and obstetric haemorrhage accounts for more than one quarter of maternal deaths [7, 8]. The Millennium Development Goals aimed to reduce maternal mortality by three-quarters by 2015. The World Health Organization (WHO) indicated that reducing deaths from PPH by 75% would enable this target to be achieved [8, 9]. Many Sub-Saharan countries unfortunately failed to achieve the goal by the end of 2015, and the annual number of maternal deaths remains very high [8]. The United Nations has launched a global strategy (2016–2030) for women’s, children’s and adolescent’s health to complete the unfinished work of the Millennium Development Goals. The strategy aims to address inequities within and between countries and to help countries begin implementing the 2030 Sustainable Development Goals in which one of the specific objective is to reduce the global MMR to less than 70 per 100,000 live births [10].

Maternal deaths due to PPH have increased in many countries and underlying factors such as increasing maternal age, caesarean section rates and multiple pregnancies have been identified [1, 11–15]. Women in low and middle income countries have an increased likelihood of severe haemorrhage and of dying from PPH-related complications [16].

In Namibia, the MMR increased from 338 maternal deaths per 100,000 live births in 1990 to 390 maternal deaths per 100,000 live births in 2005 [17]. However, the MMR has subsequently improved and was reported as 265 per 100,000 live births in 2015, although this is still far above the target of 70 per 100,000 live births [17, 18]. This means that for every 1000 live births in Namibia about three women die during pregnancy, childbirth, or within 2 months of childbirth.

According to the WHO, PPH is defined as a blood loss greater than or equal to 500 ml within 24 h after birth, while massive PPH is a blood loss greater than 1000 ml. Bleeding within 24 h after birth is considered to be primary PPH, while secondary PPH is observed from 24 h up to 12 weeks after birth [9]. Primary PPH may be caused by an atonic uterus, trauma to the birth canal as well as rare causes such as uterine inversion or clotting defects [19].

The intrapartum risk factors for PPH are induction of labour, augmentation of labour by oxytocin, prolonged or rapid labour, instrumental delivery and caesarean section. Women without risk factors can develop PPH or even conceal their bleeding internally, which is why healthcare workers should consider every woman to be at risk. More than 70% of maternal deaths due to obstetric haemorrhage are preventable and are due to substandard care [11, 15, 20].

Active management of the third stage of labour can prevent PPH. This includes actions to deliver the placenta and use of uterotonic medication within one minute of delivery, with oxytocin as the drug of choice. Controlled cord traction helps the placenta to separate from the uterus and descend into the vagina. These interventions can reduce the estimated maternal blood loss after birth by up to 66% when compared with physiological or expectant management [21].

Emergency care for PPH requires teamwork, increased awareness of the problem, and anticipatory clinical practice to prevent PPH or respond quickly and use of evidence based PPH guidelines [22]. To reduce the huge burden of PPH on maternal and child health there is a necessity to provide high quality care to women with this lethal condition. Quality care requires both facility readiness (availability of staff, key equipment, drugs supplies) and provider preparedness (knowledge and skills) for dealing with PPH [23]. Maternal deaths from PPH in Africa have been linked to poor quality of care and weak health systems. Key issues include the inability of midwives to institute life saving measures, failure to treat anaemia during antenatal care, delays in care due to poor inter-hospital transfer, difficulty accessing blood for transfusion, lack of essential medication and inadequate training of staff in obstetric emergencies [24–27].

Improving healthcare providers’ knowledge and skills in emergency obstetric care and ensuring that the healthcare facility has essential lifesaving resources, have been identified as important factors that can impact on the high MMR in many African countries [28]. Significant event analysis of maternal deaths and near-misses can also identify practical measures for tackling care deficiencies and interventions to improve maternal care quality [29–32]. No studies on the quality of emergency obstetric care, including care for PPH, have been published from Namibia.

The aim of this study was to assess and improve the quality of care for women with PPH at Onandjokwe Hospital, Namibia. The specific objectives were to assess the current quality of care for PPH; to plan and implement changes to improve the quality of care; and to assess if these changes were associated with a measurable improvement in the quality of care.

## Methods

### Study design

The study design was a quality improvement cycle (QIC) that followed six steps: the establishment of the QIC team, agreement on criteria and target standards, initial data collection, initial data analysis and feedback to the team, planning and implementation of interventions, repeat data collection and data analysis to detect change in the quality of care. Such criterion-based audit cycles have been well established as an approach to improving the quality of care [33] and have also shown particular value in reducing maternal and perinatal mortality [29]. The World Health Organization sees “clinical audit and feedback” as one of the key strategies for improving quality of clinical care and defines it as “a strategy to improve patient care through tracking adherence to explicit standards and guidelines coupled with provision of actionable feedback on clinical practice” [34].

### Setting

Onandjokwe Hospital in northern Namibia, about 750 km from the capital city of Windhoek, was the study site. The hospital provided emergency obstetric care for the Onandjokwe district, which covered approximately 25,000 km^2^ and had a population of 147,000, mostly from the Wambo ethnic group. It was also the nearest referral hospital for district hospitals in the Oshana and Ohangwena regions. There are two other such intermediate or regional hospitals in Namibia that serve their immediate communities and surrounding district hospitals.

The Department of Obstetrics and Gynaecology at Onandjokwe Hospital participated in the study. The department had a consultant obstetrician, four medical practitioners, two senior registered nurses, 26 registered nurses, six midwives and 16 enrolled nurses. The nursing staff worked in three shifts (morning shift 07h00-13h00, afternoon shift 13h00-19h00 and night shift 19h00-07h00). In 2015 there were 6407 live births at the hospital.

The maternity services had 75 beds (antenatal care 11 beds, labour ward seven beds, delivery ward four beds and postnatal ward 53 beds). The hospital had a theatre unit with two operating rooms and a functioning laboratory under the Namibia Institute of Pathology.

### Study population

The initial audit included all 82 women who delivered at Onandjokwe hospital and were diagnosed with primary PPH during 2015. The researcher identified women with PPH from the labour ward register and excluded women if they had a home delivery, developed PPH at another hospital or had a secondary PPH. The follow up audit included all 70 women who delivered at Onandjokwe and had a primary PPH between November 2016 and August 2017 (10 months). All women that presented with PPH during these time periods were included in the study and therefore there was no sampling involved within these time frames. No women were excluded from the study.

### Audit team

The main researcher (first author) led the audit team, which included two senior doctors and three nurses from the maternity services.

### Target standards

The audit team used the guideline for maternity care from South Africa and the guideline for management of PPH from South Africa to set up evidence-based criteria [35, 36]. The South African health system is similar to that in Namibia and they are neighbouring countries. The Namibian Ministry of Health and Social Services had not published a guideline on obstetric care [37]. The team agreed on measurable structural, process and outcome criteria, and set performance levels for each criterion that would be realistic goals for quality improvement in the local context.

#### Structural target standards

Structure refers to the resources needed to support quality care. These resources may be equipment, human resources or materials such as guidelines and protocols. The protocols referred to were more detailed instructions on management of conditions that were locally produced by the hospital and based on the recommendations in national guidelines. A score was used for each item: if the item existed and was functioning = 2, if the item existed but was not fully functional = 1, if the item did not exist = 0. Target standards for structure were:

- 75% of medical officers in the maternity ward were trained in obstetric emergencies and neonatal resuscitation
- 50% of nurses in the maternity ward were trained in obstetric emergencies and neonatal resuscitation
- The labour ward had a piped water supply
- There was soap for hand washing in the labour ward
- The labour ward had a functioning electricity supply
- 75% of sphygmomanometers in labour ward were functioning
- There was a functioning haemoglobin meter
- There was a guideline for maternity care in the labour ward
- There was a guideline on management of PPH
- There was standard protocol for the management of the atonic uterus
- There was standard protocol for the manual removal of placenta
- There were standard blood requisition forms
- There were informed consent forms for blood transfusion
- There were tubes for the collection of blood samples
- There were intravenous fluids (crystalloids and colloids)
- There were plastic bags for the collection of blood samples
- There were oxygen cylinders with regulators
- There were uterotonic medications (oxytocin and misoprostol)
- There were antibiotics available (β lactam and cephalosporin)

#### Process target standards

Process refers to the activities or behaviours expected of health professionals in their management of the patient. The target standards for the process were as follows:

- 80% of women were diagnosed with PPH within 45 min after delivery
- 90% of women with uterine atony were administered oxytocin within 5 min of diagnosis
- 90% of women received crystalloid fluids within 5 min of diagnosis of PPH
- 70% of women with uterine atony received uterine massage and bimanual compression if the uterus failed to contract despite oxytocin
- 50% of women with uterine atony received misoprostol within 25 min of determining failure of oxytocin
- 90% of women received prophylactic antibiotics after manual removal of placenta or uterine exploration
- 100% of women with low haemoglobin (≤6 g/dl) received blood products
- 70% of women received blood products within 15 min after being diagnosed with a massive PPH
- 75% of women with a massive PPH were managed surgically (hysterectomy) within 60 min of diagnosis or within 120 min after delivery

#### Outcome target standards

The target standards for the outcome were:

- 90% of women were treated and stabilized within 6 h of PPH
- 100% of women survived the PPH
- 70% of women on discharge were given an appointment for follow up within 1 month

### Initial data collection

The main researcher collected data from the patient’s maternity record in both the labour and postnatal wards using a standardised collection tool to measure the process and outcome criteria. The date, time of events and clinical findings were all documented in the maternity record which allowed the time taken for different interventions to be calculated. The researcher also inspected the maternity services in order to measure the structural criteria and used a simple questionnaire to evaluate the proportion of healthcare workers trained in obstetric and neonatal emergency care.

### Initial data analysis

The researcher captured the data in Microsoft Excel and checked for any omissions or errors. The researcher then used the Statistical Package for the Social Sciences version 25 to analyse the data. Frequencies and percentages described categorical data, while numerical data was summarised using means and standard deviations or median and interquartile ranges, depending on whether data were symmetrically distributed or skewed. The analysis compared results and target standards to determine the number of targets achieved at baseline.

### Planning and implementation of change

The audit team reviewed the initial data analysis and the main researcher facilitated reflection by using the “5 Whys” techniques to identify root causes of poor quality [33]. Once the team had reached a consensus on root causes their findings were presented to the Department of Obstetrics and Gynaecology for further discussion and revision. Following this, the audit team implemented changes to clinical practice over a period of 10 months (November 2016 to August 2017).

### Repeat data collection and data analysis

The researcher then collected retrospective data on the same criteria in the patients seen during the 10-month period when clinical practice was changed. Data was again analysed descriptively to see if there was improvement in the number of target standards achieved. In addition, the Pearson’s Chi-square test compared categorical data at baseline and follow up with a statistical significance at *p <* 0.05.

## Results

There were 152 files audited, 82 at the baseline audit and 70 at the re-audit. Table 1 presents a profile of the women included in the two audits. The participants’ mean age for both audits was 29 years. The two groups differed significantly in terms of their parity and marital status, with the re-audit group having more multiparous and married women, however the causes of PPH were similar between the two groups. There were three patients who had both uterine atony and vaginal tears, but the PPH was attributed to the uterine atony.

**Table 1:** Profile of mothers at baseline audit and re-audit

Variable	Baseline *N* = 82 n (%)	Re-audit *N* = 70 n (%)	*p* value
Parity			
Primiparous	34 (41.5)	18 (25.7)	0.041
Multiparous	48 (58.5)	52 (74.3)	
Marital status			
Single	62 (75.6)	36 (51.4)	0.002
Married	20 (24.4)	34 (48.6)	
Cause of postpartum haemorrhage			
Tears	17 (20.7)	12 (17.1)	0.317
Retained products of conception	13 (15.9)	18 (25.7)	
Atony	52 (63.4)	40 (57.1)	

### Structural target standards

Table 2 compares the results for structural criteria at baseline and follow up. At baseline 12/19 (63%) of target standards were achieved, while 19/19 (100%) were achieved at re-audit. The number of medical officers and nurses trained in obstetric emergencies and neonatal resuscitation increased from 2/4 (50.0%) and 1/46 (2.2%) respectively to 3/4 (75.0%) and 27/50 (54.0%), while the number of functioning sphygmomanometers increased from 1/4 (25.0%) to 4/4 (100.0%).

**Table 2:** Results for structural target standards

Structural target standards	Standard achieved	
	Baseline	Re-audit
75% of medical officers in maternity ward are trained in obstetric emergencies and neonatal resuscitation	No	Yes
50% nurses in maternity ward are trained in obstetric emergencies and neonatal resuscitation	No	Yes
The labour ward has a piped water supply	Yes	Yes
There is a soap for hand washing in labour ward	Yes	Yes
The labour ward has functioning electricity	Yes	Yes
75% of blood pressure machines in labour ward are functioning	No	Yes
There is a functioning haemoglobin meter	Yes	Yes
There is a guideline for maternity care in labour ward	No	Yes
There is a guideline on management of PPH	No	Yes
There is standard protocol for the management of the atonic uterus	No	Yes
There is standard protocol for the manual removal of placenta	No	Yes
There are standard blood requisition forms	Yes	Yes
There are informed consent forms for blood transfusion	Yes	Yes
There are cross match tubes for the collection of blood sample	Yes	Yes
There are intravenous fluids (crystalloids and colloids)	Yes	Yes
There are plastic bags for the collection of cross match sample and blood requisition forms	Yes	Yes
There is an oxygen cylinder with regulator	Yes	Yes
There are uterotonic medications (oxytocin, misoprostol)	Yes	Yes
There are antibiotics (β lactam, cephalosporins)	Yes	Yes

### Process target standards

Table 3 compares the results for process criteria at baseline and follow up. At baseline, none of the standards were achieved, but six out of nine were achieved at re-audit and six of the nine criteria significantly improved. All criteria either achieved the target standard or improved significantly.

**Table 3:** Results for process target standards

Process target standards	Baseline n (%)	Re-audit n (%)	*p* value
80% of women diagnosed with PPH within 45 min after delivery	30/82 (36.6)	55/70 (78.6)	< 0.001
90% of women with uterine atony have been administered oxytocin within 5 min of diagnosis	29/52 (55.8)	33/40 (82.5)	0.015
90% of women received intravenous crystalloid fluid within 5 min of diagnosis of PPH	38/82 (46.3)	65/70 (92.9)	< 0.001
70% of women with uterine atony received uterine massage and bimanual compression if the uterus failed to contract despite oxytocin	21/52 (40.3)	30/40 (75.0)	0.002
50% of women with uterine atony were administered misoprostol within 25 min of determining failure of oxytocin	8/39 (20.5)	28/36 (77.8)	<0.001
90% of women received prophylactic antibiotics after manual removal of placenta or uterine exploration	10/13 (76.9)	17/18 (94.4)	0.151
100% of women with low haemoglobin (≤ 6 g/dl) received blood products	54/55 (98.2)	36/36 (100.0)	0.416
70% of women received blood products within 15 min of being diagnosed with a massive PPH	13/52 (25.0)	22/36 (61.1)	0.001
75% of women with a massive PPH were managed surgically within 60 min after being diagnosed or within 120 min of delivery	2/5 (40.0)	3/3 (100.0)	0.196

### Outcome target standards

There was one maternal death at baseline and no maternal deaths at re-audit. Table 4 compares the results for the other outcome criteria at baseline and follow up. None of the standards were achieved at baseline, while two of the three standards were achieved at re-audit and two criteria significantly improved.

**Table 4:** Results for outcome target standards

Outcome target standards	Baseline *N* = 82 n (%)	Re-audit N = 70 n (%)	*p* value
90% of women treated and stabilised from complication of PPH within 6 h	60 (73.2)	64 (91.4)	0.004
70% of women were given a follow up date within one month of being discharged from hospital	21 (25.6)	43 (61.4)	< 0.001

### Changes and implementation of changes

The audit team and departmental staff identified that there was a lack of training of the professional staff in the labour ward to manage obstetric emergencies and that the staff did not have access to an evidence-based guideline and protocols. Key equipment, such as sphygmomanometers, were also not maintained or calibrated. They also identified that women with PPH were not diagnosed quickly due to a lack of effective monitoring post-partum. They made recommendations to address the poor performance observed in the baseline audit. The implementation of changes engaged all doctors and nurses involved in patient care at the maternity ward. Table 5 describes the interventions to improve care of PPH.

**Table 5:** Summary of interventions to improve care of PPH

No	Interventions
1	One hour of in-service training was organised on a weekly basis and the topic repeated for three weeks to ensure coverage of all clinical staff. The nurse in charge of the maternity ward made sure that all the nursing staff attended and expected that all doctors also attended. Topics initially focused on PPH and thereafter on a variety of obstetric topics (e.g. antenatal haemorrhage, neonatal resuscitation), including topics arising from significant events in the department. The researcher and head of obstetrics department initiated the training and subsequently different doctors and nurses also participated in leading the training.
2	Each nurse in charge of a shift made sure that all sphygmomanometers were functioning and reported any problems to the nurse in charge of maternity, who ordered new machines if necessary.
3	Guidelines and standard operating procedures were photocopied and made available for easy access to all staff members involved in patient’s management in the labour and postnatal wards
4	Nurse in charge of maternity ward allocated five beds in labour ward for women in the fourth stage of labour. An enrolled nurse took responsibility for the close monitoring of each women and recording of all clinical findings in the patient’s file. The nurse-in-charge checked all information prior to transfer to the postnatal ward. In the postnatal ward, the nurse in charge again checked the completeness of information in the maternity record (documented from labour ward) and continued the same observations and instructions

## Discussion

Significant improvements in the structure, process and outcomes of care for PPH required relatively simple interventions. After the interventions all structural standards were met and all process and outcome standards either met the target standard or significantly improved.

Structural interventions included the availability of essential equipment, clinical guidelines and the training of staff, while process interventions included the re-organisation of the labour and postnatal wards for women in the fourth stage of labour in order to monitor them closely. As a result, outcomes significantly improved such as the time taken to diagnose PPH and the percentage of women stabilised within 6-h. Reducing the time it takes to receive skilled care in an adequately resourced healthcare facility has been linked to reduced deaths from obstetric haemorrhage [28, 30].

Many countries are implementing training programmes to improve healthcare workers knowledge and skills in managing obstetric emergencies. In South Africa the Essential Steps in Managing Obstetric Emergencies (ESMOE) and the Emergency Obstetric Simulation Training (EOST) have led to significant improvement in maternity care [38]. The South African inter-ministerial committees on maternal, perinatal and child mortality have also emphasised the need to ensure 24-h access to functioning emergency obstetric care, the provision of dedicated inter-facility transport, the development of maternity homes and standardised referral criteria [39]. In Uganda interventions such as training of healthcare workers, obstetric drills and displaying guidelines was also associated with improvements in the quality of care for patients with PPH, severe pre-eclampsia and eclampsia [5].

Our study agrees with the Ugandan experience, which showed an improvement in the quality of care for PPH by the implementation of simple interventions. The sustainability of these interventions, however, requires the implementation of well-structured training programmes, equivalent to ESMOE and EOST, and strong leadership of the health system. Programmes that provide low-dose, high-frequency training in the workplace have been recommended [40] rather than longer workshops that take staff out of service delivery. ESMOE for example emphasises the need for regular “fire drills” to simulate emergency care in the clinical environment [38].

Healthcare workers diagnosed PPH by a visual estimation of blood loss. This could have been underestimated and the number of PPH cases might have been higher. The team included all women with PPH in the audits in order to minimise any sampling or selection bias and because total numbers were relatively small. The archiving of maternity records was relatively adequate, but 15 files were missing at baseline and nine files in the re-audit. It is possible that these additional files could have altered the findings if the data was available. The audit team noted one maternal death due to PPH in the baseline audit, but did not have access to the patient’s file, which was in the regional offices for evaluation.

A limitation of the QIC is that there is no control group and it is not possible to prove a causal relationship between the change to clinical practice and improvement in quality of care. Improved quality is attributed to the change in clinical practice as there is a logical relationship between the proposed cause and effect. There were no other interventions that happened outside of the QIC during this period to provide an alternative explanation for the improved quality. It is possible that similar gaps in quality exist at the other intermediate referral hospitals and that the gap is even wider at district hospitals. The quality improvement process and lessons learnt may be transferable to these other hospitals.

A QIC is a continuous process and requires a commitment of all healthcare workers and the hospital’s management to sustain and incrementally improve the quality of care over time. The completeness of information in the maternal record was part of the change to clinical practice and future audits could include a criteria to measure this. Hospitals should ensure that:

- They provide workplace-based training routinely to nursing, midwifery and medical staff and document attendance.
- Clinical guidelines and standard operating procedures are readily available on maternity wards.
- All essential equipment and resources are available, regularly checked and maintained
- Clinical practice is organised to ensure close monitoring of women post-delivery in the labour and post-natal wards
- A learning environment is created with a focus on continuous quality improvement and measurement of target standards

This research contributes to the paucity of information on quality of care within Namibia and highlights structural and process criteria that are likely to be issues at other hospitals. There is a need for health systems to incorporate such quality improvement processes at scale and not to rely on ad hoc initiatives such as this at the local level [41]. This QIC demonstrates how relatively simple interventions, that do not require substantial additional or external resources, can lead to meaningful improvement to the competence of care and better outcomes for patients. The WHO describe a range of strategies for improving clinical care that include clinical audit and feedback and suggest the need for collaborative and team-based cycles that involve multiple teams across similar hospitals [34]. In addition they suggest the need for guidelines to support clinical decision-making and care pathways as well as morbidity and mortality reviews [34]. Such guidelines are needed in the Namibian context and strategies should be embedded in a clear policy on quality improvement.

## Conclusions

The study demonstrates that the quality of care for emergency obstetrics can be improved by audit cycles that focus on the structure and process of care. The quality of care for women diagnosed with PPH in our healthcare setting was substandard and yet simple interventions led to significantly improvement. Key interventions included workplace-based training in obstetric emergencies, availability of guidelines and standard operating protocols, attention to the availability and functioning of essential equipment and medication as well as re-organisation of care to ensure close monitoring of postpartum women. Namibia should look at embedding such key strategies for quality improvement into national policy and developing collaborative team-based audit cycles.

## Data Availability

The datasets analysed during the current study are available from the corresponding author on reasonable request.

## Abbreviations

EOST: Emergency Obstetric Simulation Training
ESMOE: Essential Steps in Managing Obstetric Emergencies
MMR: Maternal Mortality Ratio
PPH: Postpartum haemorrhage
QIC: Quality improvement cycle
WHO: World Health Organization
